# Use and comparison of different internal ribosomal entry sites (IRES) in tricistronic retroviral vectors

**DOI:** 10.1186/1472-6750-4-16

**Published:** 2004-07-27

**Authors:** Victorine Douin, Stephanie Bornes, Laurent Creancier, Philippe Rochaix, Gilles Favre, Anne-Catherine Prats, Bettina Couderc

**Affiliations:** 1 Department of « Innovations thérapeutiques en Oncologie », INSERM U563, Institut Claudius Regaud, 20-24 rue du pont St Pierre, 31052 Toulouse, France; 2 Inserm U569, Institut Fédératif de Recherche Louis Bugnard, CHU Rangueil, Chemin du Vallon, 31062 Toulouse, France

## Abstract

**Background:**

Polycistronic retroviral vectors that contain several therapeutic genes linked via internal ribosome entry sites (IRES), provide new and effective tools for the co-expression of exogenous cDNAs in clinical gene therapy protocols. For example, tricistronic retroviral vectors could be used to genetically modify antigen presenting cells, enabling them to express different co-stimulatory molecules known to enhance tumor cell immunogenicity.

**Results:**

We have constructed and compared different retroviral vectors containing two co-stimulatory molecules (CD70, CD80) and selectable marker genes linked to different IRES sequences (IRES from EMCV, *c-myc*, FGF-2 and HTLV-1). The tricistronic recombinant amphotropic viruses containing the IRES from EMCV, FGF-2 or HTLV-1 were equally efficient in inducing the expression of an exogenous gene in the transduced murine or human cells, without displaying any cell type specificity. The simultaneous presence of several IRESes on the same mRNA, however, can induce the differential expression of the various cistrons. Here we show that the IRESes of HTLV-1 and EMCV interfere with the translation induced by other IRESes in mouse melanoma cells. The IRES from FGF-2 did however induce the expression of exogenous cDNA in human melanoma cells without any positive or negative regulation from the other IRESs present within the vectors. Tumor cells that were genetically modified with the tricistronic retroviral vectors, were able to induce an *in vivo *anti-tumor immune response in murine models.

**Conclusion:**

Translation of the exogenous gene is directed by the IRES and its high level of expression not only depends on the type of cell that is transduced but also on the presence of other genetic elements within the vector.

## Background

Gene therapy protocols would strongly benefit from the development of a one step technique that would allow cells to be genetically modified through the introduction of several therapeutic genes. In order to induce the translation and expression of exogenous cDNAs, carried by a single vector, researchers have cloned internal ribosomal entry sites (IRES) upstream from these exogenous cDNAs. This approach should lead to the translation of three cistrons from an unique mRNA and therefore to the consequent expression of the three encoded proteins [1-6].

In most cases, the IRES from EMCV is cloned into polycistronic vectors as it induces high levels of DNA translation [3,7,8]. The capacity of other IRESes to induce high levels of exogenous cDNA expression in different cell types has been compared to the capacity of the EMCV IRES [2-4,9-11]. However, in most cases, these comparisons were carried out after different IRESes had been inserted into a single, characterized, dicistronic (one IRES) or tricistronic (two IRESes) mRNA and after the *in vitro *vector translation efficiency had been established [9-14]. These studies are useful in choosing the IRES that will drive the *in vivo *expression of heterologous proteins, they do, however, give little information as to the potential *in vivo *interactions that occur between different IRESs cloned into the same MuLV-based retroviral vector.

We cloned tricistronic vectors encoding three different cDNAs. This involved using at least two IRESes to translate the second and third cistrons. Using the same IRES twice in a single vector could, however, induce recombination events and the loss of the second IRES and cistron. In the same way, using the same cistron twice could lead to a competition between the two IRESs for the binding to cell type specific translation factors. For these reasons, we chose to clone and compare the efficiency of different IRESes cloned into the same vector. We chose the IRES of EMCV (IRES^EMCV^), the IRES of the *c-myc *proto-oncogene (IRES^C-MYC^), the IRES of FGF-2 (IRES^FGF-2^) and the IRES of the HTLV-1 lentivirus (IRES^HTLV-1^) [1,8,15-22]. The vectors were constructed so that the third cistron was translated from the IRES^EMCV ^and the second cistron was translated from the IRES^EMCV^, IRES^C-MYC,^, IRES^FGF-2 ^or IRES^HTLV-1^.

**Figure 1:**
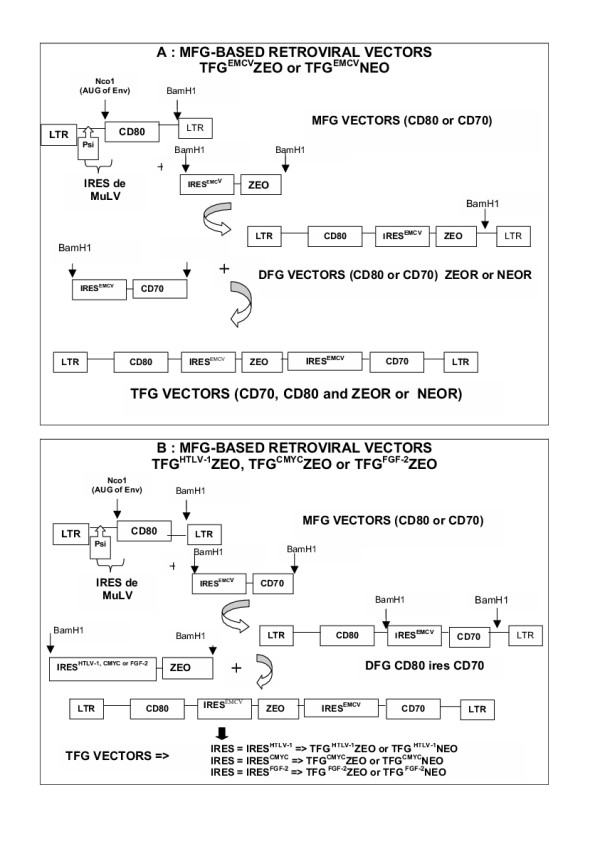
**Structure of retroviral constructs. **TFG vectors are MFG-based retroviral plasmids into which the hCD70 cDNA and hCD80 cDNAs have been cloned, with either a neoR or a zeoR selectable marker gene. The three genes are co-translated from a tricistronic mRNA which was transcribed from the 5' long terminal repeat. The translation of the first cDNA (CD70 or CD80) depends on the 5' long terminal repeat-gag IRES, while the translation of the selectable marker and the second cDNA (CD70 or CD80) are dependent on two IRESes from different origins.

Recently, it has been shown that retroviral vectors derived from MuLV contain an additional IRES on the 5' gag sequence [20,23]. The vectors described here contained three IRESes: the IRES from MuLV located between the LTR and the Psi sequence controlling the translation of the first cistron, the IRES from a different origin and the IRES from EMCV respectively controlling the translation of the second and third cistrons (Figure 1A and 1B). The exogenous genes cloned into the tricistronic vectors were chosen for their potential use in clinical trials. They code for co-stimulatory molecules known to enhance tumor cell immunogenicity: CD80, a member of the B7 family and CD70, a member of the TNF family [7,24-26]. These molecules act in synergy to enhance the induction of Ag-mediated anti-tumor immunity when co-expressed with tumor antigens [7,24,25,27]. We generated retroviral vectors that encoded the two co-stimulatory molecules CD70 and CD80, and a selection gene. We compared the efficacy of these vectors in their capacity to genetically modify various human and murine cells, and also observed how they affected the selection and culture of these cells following transduction. We then compared the expression of the three exogenous genes within the genetically modified cells. Murine melanoma cells were then tested in two different murine tumor models for their ability to induce an *in vivo *anti-tumor immune response, regardless of the percentage of co-stimulatory molecules expressed by the transduced cells.

## Results

### Construction of tricistronic retroviral vectors expressing CD70 and CD80

We constructed tricistronic vectors that would induce the expression of three cDNAs (CD70, CD80 and a selection gene) from one promoter (LTR viral promoter) (Figure 1). The constructions are described in the *Methods*. Expression of the first open reading frame (cDNA encoding a co-stimulatory molecule) occurs from one IRES (MuLV) located at the 5' extremity of the mRNA. The second and third open reading frames are translated from two identical or two different IRESes. TFG^EMCV^NEO or TFG^EMCV^ZEO were constructed so that the translation of the selection gene and the second co-stimulatory molecule could be induced from two identical IRES^EMCV ^(Figure 1A). TFG^HTLV-1^, TFG^C-MYC ^and TFG^FGF-2 ^were constructed to allow the expression of the selection gene from IRES^HTLV-1^, IRES^C-MYC ^or IRES^FGF-2 ^while the translation of the third open reading frame was under the control of IRES^EMCV ^(Figure 1B).

### Efficiency of the different IRESes in inducing the expression of the selection gene (NEO or ZEO) in different cell types

We generated retroviral vectors using the different plasmids described in Figure 1 and transfected ψCRIP cells or triple-transfected the 293T packaging cell line. We obtained viable G418 or zeocin-resistant ψCRIP cells after transfection with TFG^EMCV^NEO, TFG^EMCV^ZEO, TFG^HTLV-1^ZEO, TFG^HTLV-1^NEO, TFG^FGF-2^ZEO or TFG^FGF-2^NEO but none after transfection with TFG^cMYC^ZEO or TFG^cMYC^NEO. We tested the supernatants from ψCRIP or 293T transfected cell suspensions for the presence of replication competent retroviruses (RCR) through the dosage of reverse transcriptase activity. The supernatants from all the transfected packaging cells were free of reverse transcriptase activity. The titers of the different retroviruses produced different results, depending on the experiments, varying from 10^4 ^to 10^6 ^particles/ml. We used 10^5 ^particles of each type of retrovirus that was produced (TFG^FGF-2^ZEO, TFG^HTLV-1^ZEO or TFG^EMCV^ZEO) or the 48 hour supernatants from transiently transfected TFG^cMYC^ZEO ψCRIP or 293T cells to transduce different types of mammalian cells. The murine cells used were either NIH-3T3 fibroblasts or B16.F10 melanoma cells. To establish a human model, we cultured melanoma cells from biopsies as described in the *Methods*. During the course of this study we obtained 23 melanoma biopsies. Fifteen cell cultures were obtained from these biopsies which represents a yield of 65 %. The quality and characteristics of the melanoma cells were determined by immunohistochemistry, on cytospin cells, using anti-cytokeratin (KL-1) (negative control), anti-S100 protein, anti-melanA/MART1 and anti-HMB-45/gp100 antibody staining as described in the *Methods*. The 15 human cell cultures obtained all stemmed from melanoma cells (data not shown).

The transduced cells were selected for their resistance to G418 or zeocin. Fifteen days after the transduction with each retroviral vector, we selected G418 or zeocin-resistant cells. Apart from the TFG^CMYC^ZEO transduced cells, we obtained roughly the same number of zeocin or G418 resistant clones with the different retroviral constructs. Both murine and human cells could be successfully genetically modified using the engineered trigenic retroviral vectors. No vector was statistically more efficient in obtaining a higher yield of resistant clones. However, the TFG^EMCV^ZEO, TFG^FGF-2^ZEO and TFG^HTLV-1^ZEO cells were long lasting, viable and could be expanded, whereas neither murine nor human TFG^cMYC^ZEO transfected cells displayed long-term viability.

### The selected genetically modified cells expressed co-stimulatory molecule mRNAs

**Figure 2:**
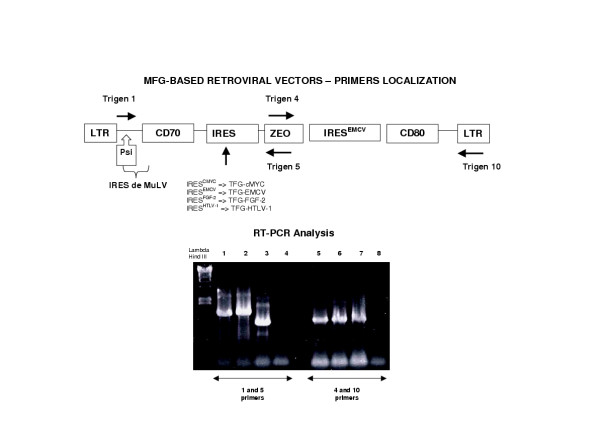
**RT-PCR analysis of mRNA transcribed from the tricistronic vectors in stably transduced melanoma cells. **2A : Localization of the different primers. Figure 2B: cDNA obtained after reverse transcription was amplified using primers 1 and 5 (lanes 1 to 4) or primers 4 and 10 (lanes 5 to 8) as described in *Methods*. Lanes 1 and 5: RT-PCR performed on mRNA isolated from TFG^FGF-2^ZEO transduced cells (1819 and 1640 bp amplified respectively). Lanes 2 and 6: RT-PCR performed on mRNA isolated from TFG^EMCV^ZEO transduced cells (1819 and 1640 bp amplified respectively). Lanes 3 and 7: RT-PCR performed on mRNA isolated from TFG^HTLV-1^ZEO transduced cells (1510 and 1640 bp amplified respectively). Lanes 4 and 8: RT-PCR performed on mRNA isolated from mock retroviral vector transduced cells (negative controls).

Cells were stably transduced with MFG derived vectors encoding CD70, CD80 and an antibiotic resistance gene. After selection with the appropriate antibiotics (G418 or Zeocin), the stably transduced cells were analyzed for the expression of the different mRNAs, by RT-PCR using specific primers. The aim of the RT-PCR analysis was to show, through the expression of the full length RNA, that successful transcription of the construct had been achieved. We were uncertain whether the transcription of the ectopic DNA was complete. RT-PCR analysis was performed using cells that were resistant to Zeocin. We are convinced that the mRNA transcribed from the ectopic DNA contains the IRES zeocin cassettes. As we can noticed in figure 2A, the primers used for PCR analysis n°4 and 5 overlapped. They hybridized with the sequence corresponding to the ZEO gene. Our hypothesis is that if we can amplify the two segments of the construction (CD70-IRES-ZEO and ZEO-IRES-CD80 or CD80-IRES-ZEO and ZEO-IRES-CD70) at the same time from the ectopic RNA, then the full length RNA had been transcribed. We have already performed other RT-PCR analyses using this the long ranger taq polymerase from Applied (France). We have been able to amplify full length RNA from all constructs (data not shown). Taken together our data strongly suggests that the full length RNA was transcribed from all the viral constructs.

These RT-PCR results suggest that there was no downregulation of CD70 or CD80 expression at the transcriptional and post-transcriptional levels.

### The selected genetically modified cells expressed the co-stimulatory molecules at different levels

**Figure 3:**
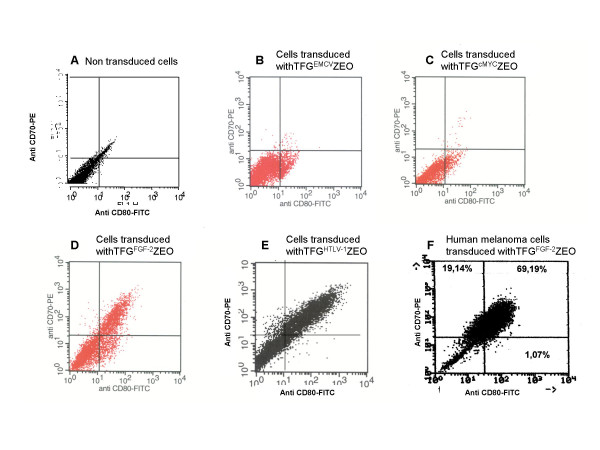
**Example of co-stimulatory molecule expression by melanoma cells after transduction with TFG tricistronic vector: Immunostaining with hCD80 –(x-axis) and hCD70 – (y-axis) 3A**: non transduced melanoma cells were cultured. Ccells (3 × 10^5^) was stained for surface expression of CD70 and CD80 using specific antibodies as described in the *Methods*. **3B-3E**: Murine B16.F10 melanoma cells were transduced with the four TFG retroviral vectors. Forty eight hours after transduction, pools of cells (3 × 10^5^) were stained for surface expression of CD70 and CD80 using specific antibodies as described in the *Methods*. 2B: TFG^EMCV^ZEO transduced cells, 2C: TFG^c-MYC^ZEO transduced cells, 2D: TFG^FGF-2^ZEO transduced cells, 2E: TFG^HTLV-1^ZEO transduced cells.**3F**: Human melanoma cells were transduced with the TFG^FGF-2^ZEO. A selected pool of cells (3 × 10^5^) was stained for surface expression of CD70 and CD80 using specific antibodies as described in the *Methods*. The samples were subjected to two-color analysis by flow cytometry. Data are shown as immunofluorescence profiles with arbitrary fluorescence units (log) of FITC on the x-axis and immunofluorescence profiles with arbitrary fluorescence units (log) of PE on the y-axis. To evaluate the percentage of co-stimulatory molecules expressed by transfected cells, the marker was set to allow < 5% positive cells in the non-transduced cells.

The cell populations were tested for co-stimulatory molecule expression by flow cytometry using Abs that were directed against CD70 and CD80. Within a given population, a large percentage of cells expressed the two co-stimulatory molecules in a stable manner and at high levels. Figure 3F shows the results of the flow cytometric analysis of human melanoma cells transduced with TFG^FGF-2^ZEO. Figure 3 is representative of the cytometric analyses carried out on the murine B16.F10 melanoma cells transduced with 48 hr supernatants of 293T cells transfected with the 4 different types of retroviral vectors. A high percentage of B16.F10 cells transduced with TFG^FGF2^ZEO or TFG^HTLV-1^ZEO retroviral vectors (Fig. 3D and 3E respectively) expressed both co-stimulatory molecules.

The percentage of B16.F10 cells, transduced with TFG^EMCV^ZEO or TFG^c-MYC^ZEO, expressing only CD80 was higher than the percentage of cells expressing only CD70 or both molecules (Fig. 3B and 3C respectively).

The level of expression of the two molecules differed depending on the vectors used and the cell types that were transduced, as shown in Table 1 - see additional file 1.

In theory, selected clones expressed both molecules. In fact, in any selected clone, we found cells that expressed only one of the molecules (CD70 or CD80) and cells that expressed the two molecules.

#### Murine cells

In murine cells (either fibroblasts or melanoma cells), a high percentage of cells only expressed CD70. This was not due to the expression of the CD70 molecule, in itself. Indeed, when we used constructs with CD80 as the first cistron, we obtained a high percentage of cells that only expressed CD80. This percentage could be attributed to a negative regulation of the translation of the third cistron.

Even after the selection of transfected cells, we obtained cells which did not express detectable levels of co-stimulatory molecules. Indeed, in TFG^HTLV-1^ZEO, TFG^FGF-2^ZEO or TFG^EMCV^ZEO transduced murine NIH-3T3 cells, we respectively found 35%, 31% or 44% of cells that expressed undetectable levels of CD70 or CD80. Expression levels were assessed regardless of the resistance gene that was used (NEO or ZEO).

We tested the expression of the two co-stimulatory molecules immediately after transduction or after selection. No correlation could be established between the time of analysis and the percentage of cells expressing both molecules. One clone could be composed of a majority of cells (60%) expressing both molecules at day 5 post selection, only have a small percentage of these cells at day 15 and up to 95 % on day 25. Indeed, even within a selected population of cells, the percentage expressing both co-stimulatory molecules could vary from 5 up to 95 % of the total cell number.

After transduction with a single vector, 20 clones were selected. Within the same clone, some cells expressed only one co-stimulatory molecule whereas others expressed both molecules. These observations are reflected by the high SD of the percentage of cells expressing co-stimulatory molecules (Table 1 - see additional file 1).

None of the vectors used were found to be adequate for the transfection of murine cells.

#### Human cells

For human cells, we chose to use a pool of transduced cells, rather than clones, to stay as close as possible to the reality of human clinical protocols. When we used TFG^HTLV-1^ZEO (CD80 first) or TFG^HTLV-1^ZEO2 (CD70 first) we obtained cells that expressed only the first cistron. We obtained a better yield of cells expressing the two co-stimulatory molecules when using the TFG^FGF-2^ZEO construct (70% of cells expressing the two molecules in a stable manner). Again, even within a selected population of cells, the percentage of cells expressing both co-stimulatory molecules could vary considerably (from 45 to 95 % of total cell number).

In human melanoma cells, the use of tricistronic vectors in which IRES^FGF-2 ^induced the translation of the second cistron, and IRES^EMCV ^the translation of the third cistron, led to a high percentage of cells expressing the three cistrons.

### Expression of the tricistronic transgene slows the tumor growth rate after s.c. injection of B16.F10 in an established model

Among selected cells, the percentage expressing both co-stimulatory molecules varied. We have previously shown that melanoma cells expressing high levels of CD70 alone or in combination with CD80 induced *in vitro *splenocyte proliferation [7]. Using a pool of selected cells that were genetically modified by TFG^HTLV-1^ZEO (52 % CD70, 21 % both CD70 and CD80), TFG^FGF-2^ZEO (5 % CD70, 33 % both CD70 and CD80), TFG^EMCV^ZEO (27 % CD70, 16 % both CD70 and CD80) or double-transfected with DFG CD70 and DFG CD80 (>65 % both CD70 and CD80), we showed that different percentages of cells expressing both co-stimulatory molecules (even as little as 20 %) could induce a proliferative response in splenocytes. A substantial increase in spleen cell proliferation was observed when the two molecules were expressed following genetic modification with all the viral vectors. This increase was similar to that induced by the use of double transfected cells (data not shown).

**Figure 4:**
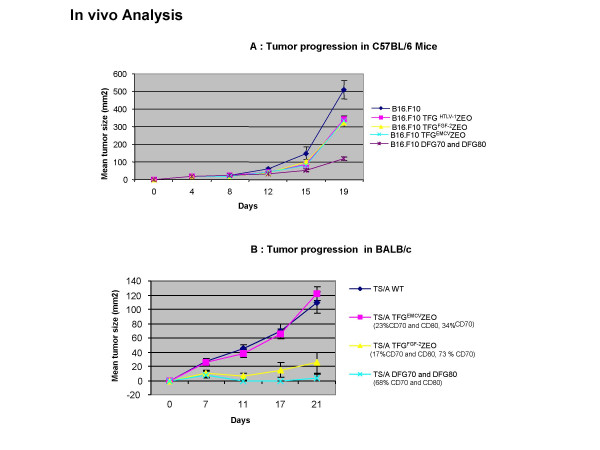
**Effects of CD70 and CD80 co-expression by single transduced tumor cells on tumor growth in an established model. 4A**: 10^5 ^B16.F10 wt (control cells), or CD70 and CD80 double transduced B16.F10, or CD70 and CD80 single transduced B16.F10 were injected s.c. on day 0 into female C57BL/6 mice. **4B**: 10^5 ^TS/A wt (control cells), or CD70 and CD80 double-transfected TS/A, or CD70 and CD80 single-transfected TS/A were injected s.c. on day 0 in female BALB/c mice. Tumor growth was monitored twice a week. The results are expressed as mean size (mm^2^) of tumors from groups that each contain five mice ± SD. The data represent mean +/- SD of a single experiment representative of three.pouet

To determine whether the local expression of CD70 and CD80 could affect tumor establishment, we sub-cutaneously injected 10^5 ^cells, from each type of tumor, into the flanks of C57BL/6 mice (Figure 4A). These cells had previously been used in splenocyte proliferation experiments. Subcutaneous injection of B16.F10 cells into B6 syngeneic immunocompetent mice led to the development of tumors. However, there was a delay in the appearance of tumors derived from transduced or double-transfected cells compared with tumors induced by the inoculation of parental or mock-transfected cells (control B16.F10 cells transfected with a retroviral vector encoding the zeocin-resistance gene). Although, all mice had palpable tumors before day 10, the growth rate was significantly slower for TFG^FGF-2^ZEO or TFG^HTLV-1^ZEO transduced cell tumors compared to parental cell tumors (p < 0,01) and double transfected cell tumors (p < 0.001) on day 20 (Figure 4A). This decrease in tumor growth was less pronounced in tumors derived from cells modified by tricistronic vectors than in tumors derived from cells modified by two independent vectors. This could be due to the level of expression of the co-stimulatory molecules on the cell surface. We have previously shown that co-expression of the two co-stimulatory molecules is necessary to induce an *in vivo *immune response. Here we show that, for the B16.F10 melanoma cell model, we had to inject as many as 6 × 10^4 ^cells (60 % of the injected cells) expressing both co-stimulatory molecules to observe an immune response.

We have previously shown that the anti-tumor effect is more difficult to obtain in a MHC class I loss variant than in a MHC class I positive model [7]. To confirm how important the percentage of cells expressing both co-stimulatory molecules is in inducing an *in vivo *immune response, we transduced TS/A adenocarcinoma cells using the same retroviral vectors, as previously described. We pooled the zeocin resistant cells and chose two pools. In the first pool of cells transduced with TFG^EMCV^ZEO, 34 % only expressed CD70 and 23 % expressed both CD70 and CD80. In the second pool of cells transduced with TFG^FGF-2^ZEO, 73 % only expressed CD70 and 20 % expressed both CD70 and CD80. These two pools were injected sub-cutaneously into syngeneic immunocompetent BALB/c mice. These injections led, in all cases, to the development of tumors. However, when the cells were double-transfected with two independent vectors and more than 70 % of cells expressed both co-stimulatory molecules, most of the palpable tumors spontaneously decreased within 10 days. In contrast, only a delay in the appearance of the tumors derived from transduced cells could be observed. This delay was more or less pronounced depending on the percentage of cells expressing both molecules. There was no delay in the appearance of tumors derived from the pool containing 34 % of cells expressing only CD70 and 23 % of cells expressing CD70 and CD80. A delay was observed in the appearance of tumors derived from the pool containing 73 % of cells expressing only CD70 and 20 % of cells expressing CD70 and CD80 (figure 4B).

## Discussion

In this study, we have constructed and tested different tricistronic retroviral vectors containing IRES elements from different origins. As IRESes are remarkably efficient when used in bicistronic vectors, it was particularly interesting from a biotechnological point of view (for gene therapy protocols) to design polycistronic vectors that could allow the expression of several proteins from the same mRNA. Several authors have reported the construction of different vectors using the IRES^EMCV ^or the IRES^FMDV2A ^to trigger the high level expression of an exogenous gene[2-4,10,11,28]. Prats and coll. have described several other IRESes including the IRESs from the *c-myc *proto-oncogene, the IRES from FGF-2 and the IRES from HTLV-1 [17,18,20,21]. The first aim of our work was to determine whether one of these IRESes could induce a higher level of exogenous protein expression than the IRES from EMCV in different cell lines. The second aim was to design polycistronic vectors carrying different IRESes to avoid the risk of recombination between IRESes. The third aim was to obtain genetically modified melanoma cells through transduction with tricistronic retroviral vectors. These tumor cells would therefore be genetically modified to express two co-stimulatory molecules, CD70 and CD80, which are known to induce an anti-tumor response in syngeneic mice.

As we have generated retroviral vectors derived from the well known MFG vector (MuLV vector), we already had one additional IRES upstream from the first ATG codon (initiation codon of the first exogenous cDNA). This IRES has been described by Vagner and coll [20]. The first IRES (MulV) and the third IRES (EMCV) were conserved in all the vectors. The second IRES responsible for inducing the translation of the selectable marker, was the IRES from either EMCV, FGF-2, *c-myc *or HTLV-1.

The choice of IRES that was used to express the cDNA encoding the G418 or zeocin-resistance genes was unimportant as we obtained resistant cells in all the cell lines tested: murine tumor cells (B16.F10, TS/A), murine fibroblasts (NIH3T3), packaging cell line (ψCRIP), human melanoma and lung adenocarcinoma cells (A549). However, when the IRES from the *c-myc *proto-oncogene was used, we never obtained long-lasting zeocin or G418 resistant murine or human cells, whether these were tumor cells or fibroblasts. So far, most of the cellular mRNAs that contain IRESes and code for proteins involved in the control of cell proliferation and differentiation, require stringent regulation like for example the *c-myc *mRNAs [13,15,29,30]. These genes need to be expressed at very specific stages of the cell cycle and/or in response to different stimuli [17,19]. This has also been shown for FGF-2. Indeed the CUG-initiated isoforms of FGF-2 are translationally activated in response to stress [21]. Such observations suggest that when the IRES^c-myc ^is used, translation is strongly downregulated [30].

We obtained long-lasting viable resistant cells when we used the IRESes from EMCV, FGF-2 or HTLV-1. The number of clones obtained after transduction or transfection was roughly the same depending on the experiments and the cell lines tested. This indicated that only a few (if any) recombination events occurred when we used the same IRES (EMCV) twice in the same retroviral vector.

The first and third IRESes are responsible for inducing the translation of the two co-stimulatory molecules. These IRESes competed to induce the expression of the two exogenous cDNAs. Indeed within the same population of selected cells, whatever the retroviral vector used, we were able to obtain cells that only expressed the first exon, or only the third exon or both exons. The percentage of cells that expressed both co-stimulatory molecules varied with the cell passage and from one selected clone to another. This is true regardless of the cell line tested: murine tumor cells (B16.F10, TS/A), murine fibroblasts (NIH3T3), murine packaging cell line (ψCRIP), human melanoma cells and human lung adenocarcinoma cells (A549). There is a difference in the IRES-dependent mechanism that occurs in cellular and viral internal initiation. Currently, two cellular *trans*-acting factors, the La antigen and PTB have been found to bind to picornavirus IRES elements and to be essential for their internal initiation of translation [29,31,32]. However, these proteins do not specifically bind to eukaryotic cellular mRNAs with the same efficiency. IRES function must require either different amounts of translation initiation factors or, more likely, additional proteins similar to those required for the cap-dependent initiation of protein synthesis [29,33]. Borman and coll. have recently shown that the recognition of different IRES elements varies within different tissue culture cell lines [9,12]. The activity of a particular IRES within a cell may be dependent on the relative level of stimulatory and inhibitory molecules [34]. It is possible that different *trans*-acting factors that are dependent on a specific IRES may be required. It could be that the MuLV IRES (first exon) binds those *trans*-acting factors with a higher affinity than the IRES from EMCV (third exon). Anthony and Merrick suggested that translation factors, that have a higher affinity for the cap structure than for the IRES element, could be sequestered at the m7GpppN cap structure and would therefore be unavailable for the internal initiation of translation at saturating concentrations of capped bicistronic RNA [1,35]. This would lead to an increased translation of the first cistron and a decreased translation of the second and third cistrons that are thought to be dependent on internal initiation of translation. In our case, we have an IRES (IRES MulV) on the 5' end of the mRNA. This IRES induces the translation of the first co-stimulatory molecule. However, while the IRES from EMCV or HTLV-1 could interact with other IRESes present within the retroviral construct (the IRES from gag or the IRES from EMCV), we found that the IRES from FGF-2 induced the expression of exogenous cDNA in human melanoma cells without any positive or negative regulation from the other IRESes.

We have previously shown that two co-stimulatory molecules (CD70 and CD80), expressed on the surface of tumor cells could induce an anti-tumor immune response when the cells were injected into a syngeneic animal [7,27]. In tumor cells that were genetically modified by the tricistronic retroviral vector, we attempted to induce an *in vivo *anti-tumor response in murine models. We first showed that B16.F10 cells that were genetically modified by the tricistronic vectors, could induce *in vitro *proliferation of spleen cells. These B16.F10 cells were then injected into syngeneic animals and tumor growth was monitored. We observed that in this murine model (C57BL/6) or in the BALB/c murine model (breast adenocarcinoma TS/A cells), co-expression of the two co-stimulatory molecules by at least 50 % of cells was necessary to induce an anti-tumor response. CD70 expression, alone or in association with a low level of CD80 expression, was not sufficient to induce anti-tumor immunity. These findings show that at least 50 % of the genetically modified cells must express the CD70 and CD80 co-stimulatory molecules before starting an immunotherapy protocol. Establishing cultures of human melanoma cells derived from biopsies was very difficult, we obtained a low yield of 63 %. We could infect human melanoma cells with the tricistronic retroviral vector with an efficiency of 50 %. However, within the 50 % of cells that were genetically modified we could only obtain a high percentage of cells expressing both co-stimulatory molecules when we used the tricistronic recombinant amphotropic viruses obtained with the IRES FGF-2.

## Conclusion

The ability of retroviral vectors carrying IRESes to deliver genes, *in vitro *and *in vivo, *to a variety of dividing cell types has been applied to research and gene therapy for the past 10 years. Our work shows that it is difficult to chose which IRES must be inserted into a polycistronic gene therapy vector, when the aim is to ensure a high level of translation of the exogenous gene. This level of expression will depend on the type of cell that is transduced but also on the presence of other genetic elements within the vector.

## Methods

### Cell lines

The murine melanoma B16.F10 cell line and the mouse mammary adenocarcinoma TS/A cell line, previously described, were cultured in RPMI 1640 supplemented with 2 mM glutamine and 10% fetal calf serum (FCS) (GIBCO-BRL, Cergy-pontoise, France). NIH-3T3 cells and the cysteine-rich intestinal protein (ψCRIP) and 293T packaging cell lines, were purchased from the American Type Culture Collection (Rockville, Md, USA). These three cell lines were cultured in Dulbecco's modified Eagle's medium supplemented with 10 % FCS [27]. All cell lines were periodically tested for mycoplasma infection using a DNA hybridization probe (Stratagene, La jolla, CA, USA).

### Primary culture of human melanoma cells

Melanoma tumor biopsies were dissected into small explants and then enzymatically digested with collagenase (5 mg/ml) and hyaluronidase (3 mg/ml) (SIGMA, Saint Quentin Fallavier, France) for 1 hour at 37°C under agitation. The cells were then centrifuged (5 minutes at 1200 rpm) and transferred to a culture flask and left to proliferate for 10 days. These cells were cultured in RPMI 1640 supplemented with 2 mM glutamine, 10% FCS, non-essential amino acids and vitamins (SIGMA, France). During the first 4 days after the first passage, cells were cultured with 0.1 mg/ml of G418 (GIBCO-BRL, Cergy-pontoise, France) to remove fibroblasts as described by Mouriaux and coll[36]. The quality and characteristics of the melanoma cells were studied by immunohistochemistry using anti-cytokeratin (KL-1), anti-S100, anti-HMB45/gp100 and anti-melanA/MART1 (clone A103) antibodies (all from DAKO SA, Trappes, France).

### Retroviral constructs

The pMDgag/pol and pBA-GALV plasmids were obtained from the Genethon (Evry, France). The tricistronic vectors are MFG-based retroviral vectors. These vectors were derived from the DFG-human-CD80 (hCD80) and DFG-human-CD70 (hCD70) vectors that have previously been described [7,27]. The first cistron (cDNA encoding hCD70 or hCD80) was cloned between the Nco1 and BamH1sites of the MFG vector. The AUG of this molecule corresponds to the AUG of the *env *gene of the MFG vector.

#### Construction of TGF^EMCV^ZEO or NEO

In these vectors, the translation of the genes encoding the two co-stimulatory molecules and the cDNA encoding the G418 or zeocin resistance genes are under the control of the IRES^EMCV^. We digested the Blue-script plasmid (PKs) (Stratagene, La jolla, CA, USA) with Xho1 and EcoRV to insert the cDNA encoding IRES^EMCV ^(636 bp), obtained from PCR amplification of pIRES-EGFP (Clontech, Palo Alto, CA, USA), and obtained a PKs IRES^EMCV ^vector. This vector was digested by Nco1 and EcoR1 and ligated with the cDNA encoding either CD70 or CD80, obtained from the PCR amplification of DFG-CD70 or DFG-CD80 respectively. The PKs IRES^EMCV ^CD70 or PKs IRES^EMCV ^CD80 vectors were digested by BamH1. These inserts have been cloned into either DFG CD80 NEO or ZEO by partial digestion using BamH1, generating the (T) tricistronic vectors TFG^EMCV^NEO or TFG^EMCV^ZEO with CD80 as first cistron and CD70 as third cistron or in DFG CD70 NEO or ZEO generating the same type of tricistronic vectors but where CD70 is the first cistron and CD80 is the third cistron.

#### Construction of TGF^FGF-2^ZEO, TGF^HTLV-1^ZEO, TGF^C-MYC^ZEO or NEO

Through PCR amplification, we cloned the DNA encoding the IRES of the human basic Fibroblast Growth factor (FGF-2) (547 bp), the IRES of the *c-myc *oncogene (cMYC)(602 bp) and the IRES of the HTLV-1 lentivirus (249 bp) upstream from the cDNA encoding the G418 or zeocin resistance genes in the PKs plasmid. We generated different combinations of cDNAs with BamH1 sites on both the 5' and 3' ends: IRES^HTLV-1^-NEO, IRES^HTLV-1^-ZEO, IRES^FGF-2^-NEO, IRES^FGF-2^-ZEO, IRES^cMYC^-NEO and IRES^cMYC^-ZEO. These constructs were inserted downstream from the gene encoding the first co-stimulatory molecule on the BamH1 site. We generated the TFG^FGF-2^NEO or ZEO, or TFG^c-MYC^NEO or ZEO and TFG^HTLV-1^NEO or ZEO tricistronic vectors (Figure 1A). All the vectors were sequenced.

### Transfection and transduction

CRIP packaging cells were transfected with the different plasmids using the lipofectamine technique followed or not by selection with G418 (1 mg/ml) (from Life Technologies, GIBCO-BRL, France) or Zeocin (0.2 mg/ml) (CAYLA, France). The resistant CRIP clones were expanded and screened for viral titers which were of approximately 10^4 ^viral particles/ml/48 hours/10^6 ^CRIP cells. 293T cells were triple-transfected with the different plasmids: TFG, pMDgag/pol and pBA-GALV. Estimation of the viral titer in transiently transfected 293T cells showed the presence of 10^6 ^viral particles/ml/48 hours/10^6 ^293T cells. Fibroblasts or tumor cells (B16.F10, TS/A or human melanoma) were transduced with the retroviral vectors using polybrene (8 μg/ml) (SIGMA, France). The stably transduced cells were selected using G418 or Zeocin depending on the viral particles used, and the resistant cells were either cloned or pooled and used in subsequent experiments.

### RT-PCR

Cells were cultured in T-75 cm^2 ^culture-flasks for 72 h until they reached sub-confluence. Total RNA was isolated from a suspension, as described by Choczynski and Sacchi (1987) [37], of 5 × 10^6 ^cells using TRIZOL™ reagent following the manufacturer's recommendations (Life Technologies, Cergy Pontoise, F). RNA solutions were treated with 5 units of DNAse 1 (Roche Diagnostics, Mannheim, D) to remove any contaminating genomic DNA. mRNA was transcribed into cDNA using the Ready-to-go™ kit (Amersham Pharmacia Biotech. Inc. Piscataway, NJ, USA) and random primers were purchased from Life Technologies. Amplification of cDNA was carried out using 1U/100 μl of Taq DNA polymerase (Roche Diagnostics, Mannheim, D) in a PTC-100™ Programmable Thermal Controller (MJ Research, Watertown, Mass, USA) after 33 temperature cycles consisting of denaturation at 94°C (60 s), annealing at 65°C (60 s) and elongation at 72°C (120 s). The following primers were used: MFG sense primer (Trigen 1): 5'-TGTAAAACGACGGCCAGTCACGTGAAGGCTGCCGACC-3', ZEO anti-sense primer (trigen 5): 5'-CAGGAAACAGCTATGACCCACCGGAACGGCACTGGTC-3', ZEO sense primer (trigen 4): 5'-TGTAAAACGACGGCCAGTGACCAGTGCCGTTCCGGTG-3' and MFG anti-sense primer (trigen 10): 5'-CAGGAAACAGCTATGACCGCCTGGACCACTGATATCCTGTC-3'. PCR products and the lambda/hindIII molecular weight marker (Promega, Lyon, F) were separated by electrophoresis on 0,8% agarose (Roche, Mannheim, D), Tris Borate EDTA (Interchim, Monluçon, F) gels and visualized by staining with ethidium bromide.

### Immunostaining and flow cytometric analysis

Transduced cells were stained for membrane expression of the two co-stimulatory molecules using PE-conjugated mouse anti-human CD70 mAb and FITC-conjugated mouse anti-human CD80 mAb (Pharmingen, Hamburg, D) as previously described [7]. Stained cells were analyzed using a FACScalibur (Becton Dickinson, Mountain View, USA).

### Establishment of murine tumor models

Female C57BL/6 (H-2b), or BALB/c (H-2d) mice were obtained from CERJ Janvier (St Quentin-Fallavier, France). All ear-tagged mice were kept in the special pathogen-free animal facility in our institution and were used for experiments between the age of 6 to 8 weeks. To establish subcutaneous (s.c.) tumors, 105 cells (twice the minimal tumorigenic dose) were suspended in 0.1 ml of PBS and injected s.c. into the flank. Animals were examined daily until the tumor became palpable, the diameter of the tumor, in two dimensions, was then measured twice a week. The animals were sacrificed when the tumor size reached 2.5 × 2.5 cm in the control group. The statistical significance of the data was established using the two sample student's t-test. A p-value of less than 0.01 was considered to be statistically significant.

## Authors' contributions

VDE carried out the cellular and *in vivo *studies, SB the synthesis of the retroviral vectors (molecular biology and virology). PR performed the characterization of the melanoma biopsies. LC provided all the IRES sequences. BC and ACP are responsible for conceiving this work. BC participated in the design, coordination and drafting of this manuscript. GF is the director of the laboratory. All authors read and approved the final manuscript.

## Supplementary Material

Additional file 1 Click here for file
