# Supplementary material for: Use and comparison of different internal ribosomal entry sites (IRES) in tricistronic retroviral vectors
Source: BMC Biotechnol. 2004 Jul 27;4:16. doi: 10.1186/1472-6750-4-16 (PMC514710; doi:10.1186/1472-6750-4-16)
Supplement: Additional file 1 [file 1472-6750-4-16-S1.doc]

# TABLE 1

| CELLS | Vectors used for  Transduction or  Transfection | Percentage of cells  Expressing  Only CD70 | Percentage of cells  Expressing  Only CD80 | Percentage of cells  Expressing  Both CD70 and CD80 |
| --- | --- | --- | --- | --- |
| Murine  NIH-3T3 | TFG^HTLV-1^ZEO  TFG^FGF-2^ZEO  TFG^EMCV^ZEO | 20% ± 20%  51 % ± 21 %  10 % ± 8 % | 9 % ± 10 %  5 % ± 4 %  25 %+ 17 % | 36 % ± 14 % (MFI 231 and 208 respectively)  13 % ± 8 % (MFI 154 and 165 respectively)  21 % ± 11 % (MFI 87 and 123 respectively) |
| Murine  B16.F10 | TFG^HTLV-1^ZEO  TFG^FGF-2^ZEO  TFG^EMCV^ZEO | 48 % ± 28 %  10 % ± 7 %  26 % ± 8 % | < 1 %  3 % ± 2 %  < 1 % | 20 % ± 9 % (MFI 198 and 150 respectively)  31 % ± 3 % (MFI 151 and 264 respectively)  15 % ± 11 % (MFI 76 and 102 respectively) |
| Human  Melanoma  Cells | TFG^HTLV-1^ZEO  TFG^FGF-2^ZEO  TFG^EMCV^ZEO | 41% ± 16 %  20 % ± 4 %  43 % ± 10 % | < 1 %  < 1 %  < 1 % | < 1 %  70 % ± 9 % (MFI 210 and 190 respectively)  44 % ± 12 % (MFI 95 and 107 respectively) |

# Expression the co-stimulatory molecules by the stably transduced murine or human melanoma cells

The cells were transduced with TFG^HTLV-1^ZEO or NEO, TFG^FGF-2^ZEO or NEO or TFG^EMCV^ZEO or NEO. Selected clones (3 x 10^5^ cells) were stained for the surface expression of CD70 and CD80 using specific antibodies as described in Materials and Methods and as shown in Figure 2 at different time post transduction. The samples were subjected to two-colour analysis by flow cytometry. This table shows the percentage of cells expressing one or two of the co-stimulatory molecules.

MFI : Mean Fluoresence Intensity.
